# Farm-Gate Prices as an Economic Signal of Foot-and-Mouth Disease Vaccination Performance in Hanwoo Cattle: A Nationwide Time-Lag Analysis

**DOI:** 10.3390/vaccines14090743

**Published:** 2026-08-27

**Authors:** Dae Sung Yoo, Yoonyoung Choi, Ji-Hyun Son, Ho-Seong Cho, Yeonsu Oh

**Affiliations:** 1 College of Veterinary Medicine and Animal Medical Institute, Chonnam National University, Gwangju 61186, Republic of Korea; shanuar@chonnam.ac.kr; 2 FMD and Large Animal Health Control Division, Ministry of Agriculture, Food and Rural Affairs, Sejong 30110, Republic of Korea; yychoi82@korea.kr (Y.C.); jhsohn@korea.kr (J.-H.S.); 3 College of Veterinary Medicine and Bio-Safety Research Institute, Jeonbuk National University, Iksan 54596, Republic of Korea; hscho@jbnu.ac.kr; 4 College of Veterinary Medicine and Institute of Veterinary Science, Kangwon National University, Chuncheon 24341, Republic of Korea

**Keywords:** foot-and-mouth disease, vaccination, post-vaccination monitoring, seropositivity, livestock economics, Hanwoo, time-lag analysis

## Abstract

**Background/Objectives**: In mandatory livestock vaccination programs, post-vaccination immunity depends on both vaccine characteristics and consistent on-farm implementation. We evaluated whether Hanwoo farm-gate prices were temporally associated with subsequent foot-and-mouth disease (FMD) structural protein (SP) antibody seropositivity, used as a population-level proxy for vaccination performance. **Methods**: We analyzed 75,408 individual SP antibody records from Hanwoo cattle on commercial farms (≥50 head) sampled on-farm from April 2019 to December 2022 and linked monthly seropositivity to four price metrics. A separate 2022 cohort (*n* = 131,805) was analyzed by multivariable logistic regression. Cross-correlations assessed price-leading lags of 0–12 months; linear and exploratory hinge models evaluated association magnitude and a candidate breakpoint. **Results**: Overall SP seropositivity was 96.3%. Cattle from commercial farms had higher odds of antibody negativity than those from smaller farms (adjusted odds ratio [OR] 1.15, 95% confidence interval [CI] 1.05–1.26), whereas each additional recorded vaccination was associated with lower odds (OR 0.89, 95% CI 0.88–0.90). Monthly price and the 3-month moving-average price showed their strongest associations with subsequent seropositivity at 7 and 6 months, respectively (r = 0.519 and 0.506; both *p* < 0.001). Month-to-month price changes were not associated with seropositivity. An exploratory hinge model identified a candidate breakpoint near KRW 6.26 million per 600 kg live animal. **Conclusions**: Sustained farm-gate price conditions, rather than short-term volatility, were associated with subsequent FMD post-vaccination seropositivity. Livestock price data may provide a contextual signal for targeting communication and vaccination support activities; however, this ecological association does not demonstrate price-driven behavioral change at individual farms, and the candidate breakpoint requires prospective validation.

## 1. Introduction

Foot-and-mouth disease (FMD) is among the most economically consequential transboundary animal diseases, and vaccination is central to its prevention and control in endemic and high-risk settings [1,2]. Following the large 2010–2011 epidemic, the Republic of Korea implemented mandatory routine FMD vaccination for cattle and strengthened nationwide post-vaccination monitoring [3,4]. Under the Korean program, cattle receive an initial vaccination series followed by routine boosters at approximately 6-month intervals, while year-round serological surveillance and vaccination records are used to assess program performance [4,5]. Nevertheless, mandatory coverage does not guarantee uniform biological protection. Korean surveillance has continued to identify settings with insufficient antibody positivity, indicating that legal compliance, correct vaccine delivery, and measurable post-vaccination immunity are related but distinct components of program effectiveness [6].

On-farm vaccination is therefore not a single technical act but a recurring management process involving vaccine procurement, storage, scheduling, animal restraint, injection, and record keeping. Across livestock systems, producers’ preventive decisions are shaped by perceived disease risk, confidence in the effectiveness of recommended measures, and expectations of their benefits [7,8,9,10,11,12,13,14,15]. Economic feasibility, labor constraints, and access to veterinary information and services further influence whether such measures are adopted and sustained [16,17,18,19,20]. International studies illustrate these contextual influences in different production systems: FMD control intentions and veterinary vaccine adoption have been examined in Ethiopia [9,19], the farm-level economics of routine FMD vaccination in South Vietnam [16], participation in livestock vaccination programs in India [17], and farmer-oriented delivery of veterinary services in Kenya [20]. The practical quality of implementation is equally important. Field studies have documented deficiencies in injection technique and training [21], exposure of veterinary vaccines to inappropriate on-farm storage temperatures [22,23], and substantial variation in the feasibility and uptake of disease prevention measures among farms [24,25,26]. Recent post-vaccination work in Korean cattle has likewise suggested that farm infrastructure, including the availability of suitable restraint facilities, may influence the consistency of vaccine administration and the resulting serological response [27].

Taken together, these findings point to three distinct levels at which a vaccination program can succeed or fail. First, vaccine-related factors determine whether an appropriately administered dose is capable of generating an adequate immune response [2,4]. Second, delivery-related factors—including storage, handling, injection technique, animal restraint, and booster timing—determine whether that biological potential is realized under field conditions [21,22,23,27]. Third, management and behavioral factors determine whether recommended practices are implemented consistently over time [7,8,9,10,11,12,13,14,15,16,17,18,19,20,24,25,26]. These levels are often evaluated separately, although post-vaccination serological surveillance reflects their combined effects. From a programmatic perspective, routinely available contextual indicators that anticipate periods of lower serological performance could therefore complement conventional monitoring, provided that such indicators are not interpreted as direct measures of farmer compliance.

Despite this evidence, evaluations of national vaccination programs commonly treat vaccination as a fixed input, with less attention to the farm management conditions that may generate temporal variation in implementation [28,29]. Farm size may be relevant because larger operations require more complex scheduling, batch handling, delegation of injections, and coordination of labor, although the direction of its association with preventive practice is not uniform across production systems [13,24,25,26,27,30,31]. Hanwoo, the predominant native beef breed in Korea, is raised in operations ranging from small owner-managed herds to large commercial farms. National farm-gate prices for a standardized 600 kg live animal are continuously reported and provide a readily available measure of the market environment experienced by producers. In this study, “farm-gate price” refers to the producer-level market price of Hanwoo cattle, not the price of the FMD vaccine. Evidence from beef cattle producers in the United States indicates that willingness to invest and engagement with veterinary services are associated with stronger disease prevention preparedness [32], but direct evidence linking livestock output prices with subsequent vaccination performance remains limited.

Most published evidence linking economic conditions to livestock disease prevention has focused on farm-level decisions rather than longitudinal immune outcomes. Previous studies have evaluated the benefit–cost of FMD vaccination [16], farmers’ intentions or participation in vaccination programs [9,17,19], constraints to biosecurity implementation [18], and the accessibility and design of veterinary services [20]. These studies demonstrate that prevention is embedded within an economic and service delivery context, but they do not establish whether routinely observed livestock market conditions are temporally associated with measured post-vaccination immunity. The Korean system provides a distinct setting for this question because FMD vaccination is mandatory and serological monitoring is conducted routinely. Accordingly, variation in SP seropositivity is better interpreted as variation in population-level vaccination performance than as a simple measure of vaccine uptake, creating an opportunity to examine whether a continuously reported market indicator adds contextual information to surveillance.

The national FMD monitoring system routinely measures antibodies to viral structural proteins (SPs). In a population subject to mandatory vaccination and with limited field-virus circulation, monthly SP seropositivity is a useful population-level indicator of vaccination performance; however, it is not a direct measure of farmer behavior. An antibody-negative result may reflect missed or incomplete vaccination, improper storage or administration, the timing of sampling, or individual immunological non-response [4,6,21,22,23,27,33]. Against this background, this study tested whether sustained changes in the farm-gate price of Hanwoo cattle preceded changes in post-vaccination SP seropositivity. We also examined cattle-level factors associated with SP antibody negativity and explored whether the price–seropositivity relationship showed a candidate breakpoint. We hypothesized that lower sustained farm-gate prices would be followed by lower population-level seropositivity after an operationally plausible time lag.

## 2. Materials and Methods

### 2.1. Study Design and Data Sources

A retrospective nationwide epidemiological study was conducted by integrating FMD post-vaccination serological monitoring records with monthly Hanwoo farm-gate price data. Individual cattle-level SP antibody records were obtained from the national post-vaccination monitoring database operated by the Animal and Plant Quarantine Agency (APQA). Available variables included sampling date, most recent vaccination date, cumulative number of recorded vaccinations, herd-size class, sampling site, and the binary SP antibody result. Monthly farm-gate prices for a standardized 600 kg live-weight Hanwoo animal were obtained from the Korea Institute for Animal Products Quality Evaluation (KAPE) and the Korea Agricultural Marketing Information Service (KAMIS). Price observations from January 2018 onward were retained to construct moving-average and lagged exposure metrics for the serological analysis period.

The study comprised two complementary analytical components using different units of analysis. The first was a cattle-level risk factor analysis in which the outcome was the binary SP antibody result for individual animals. The second was an ecological time-lag analysis in which eligible animal records were aggregated by calendar month and compared with national monthly Hanwoo price series. These components addressed related but distinct questions: the cattle-level analysis characterized factors associated with antibody-negative status, whereas the ecological analysis evaluated whether changes in the market environment preceded changes in population-level seropositivity. The two components were therefore interpreted together but were not treated as a single multilevel or causal model.

### 2.2. Study Populations and Eligibility Criteria

Two analytical populations were used. First, a 2022 cattle-level risk factor cohort was analyzed to identify factors associated with SP antibody-negative status. The model included cumulative number of vaccinations, herd size class (commercial farms with ≥50 head versus farms with <50 head), sampling site (on-farm, slaughterhouse, or stud farm), and the interval from the most recent vaccination to antibody testing.

Second, the time-lag cohort was restricted to Hanwoo cattle from commercial farms (≥50 head) sampled on-farm. To reduce heterogeneity attributable to maternal antibodies and extensive booster histories, animals were required to be younger than 24 months and to have received four or fewer cumulative vaccinations. Records were excluded when the interval between the most recent vaccination and sampling was <14 days. Because an emergency nationwide vaccination campaign was conducted in early 2019, the principal serological time series was restricted to April 2019–December 2022, yielding 45 monthly observations.

### 2.3. Outcome and Price Exposure Metrics

The individual outcome was a binary SP antibody result. For the time-lag analysis, eligible records were aggregated by calendar month, and monthly population-level seropositivity was calculated as the number of SP antibody-positive cattle divided by the total number tested. Four price exposure metrics were constructed: the monthly farm-gate price, month-to-month percentage change (PCT), the 3-month moving average (MA3), and the 6-month moving average (MA6). Prices were expressed as KRW per standardized 600 kg live animal.

For month t, the month-to-month percentage change was calculated as 100 × [P(t) − P(t − 1)]/P(t − 1), where P(t) denotes the monthly farm-gate price. The MA3 and MA6 variables were trailing arithmetic means of the current and preceding 2 or 5 monthly prices, respectively. The four exposure metrics were selected to distinguish the absolute market level, short-term volatility, and more sustained price conditions. For lagged analyses, each price metric was aligned with a later monthly seropositivity observation so that a positive lag indicated that the price exposure occurred before the antibody outcome.

### 2.4. Statistical Analysis

Multivariable logistic regression was used to estimate adjusted odds ratios (ORs) and 95% confidence intervals (CIs) for SP antibody-negative status in the 2022 cohort. For the ecological time-lag analysis, Pearson cross-correlation coefficients were calculated between each price metric at month t − k and monthly SP seropositivity at month t for lags k = 0, 1, …, 12 months. A positive lag therefore indicates that price preceded the antibody outcome. The relationship between the MA3 price at a 6-month lag and seropositivity was further summarized using ordinary least-squares regression. An exploratory hinge model, y = β0 + β1 max(x − τ, 0), was fitted by grid search to identify a candidate price breakpoint τ; its 95% CI was estimated using 200 bootstrap replicates. Statistical significance was defined as *p* < 0.05. Because the lag analyses were hypothesis-generating, *p*-values were not adjusted for multiple lag comparisons; interpretation therefore emphasized the magnitude, timing, and coherence of the pattern across related price metrics rather than any isolated *p*-value. Monthly cross-correlations assigned equal weight to each calendar month, although the number of cattle tested varied among months. This unweighted approach treated calendar month as the ecological unit of analysis and was intended to characterize temporal co-movement between monthly summaries rather than estimate an individual-animal effect. Consequently, monthly seropositivity estimates from months with fewer tests were less precise, which was considered a limitation. The bootstrap CI for the hinge model quantifies uncertainty conditional on the selected MA3/6-month-lag specification; it does not incorporate uncertainty arising from alternative lag or moving-average choices or from the multiple lag comparisons. Data preprocessing was conducted in R version 4.3 [34], and modeling and visualization were implemented in Python version 3.10.

The 0–12-month lag range was selected to examine associations from the concurrent month through one year and to include the approximately 6-month booster interval used in the Korean cattle vaccination program. For the ordinary least-squares model, each monthly observation contributed one price–seropositivity pair. The hinge model was used only as an exploratory description of possible nonlinearity: below the candidate breakpoint τ, the fitted contribution of price was constant, whereas above τ, the fitted response changed linearly according to the estimated slope. Bootstrap resampling was used to quantify uncertainty around the selected breakpoint within this model specification. All analyses were interpreted as associational. No causal effect of price on farmer behavior or vaccination performance, and no validated intervention threshold, was estimated.

## 3. Results

### 3.1. Characteristics of the Commercial Hanwoo Cohort

**Figure 1:**
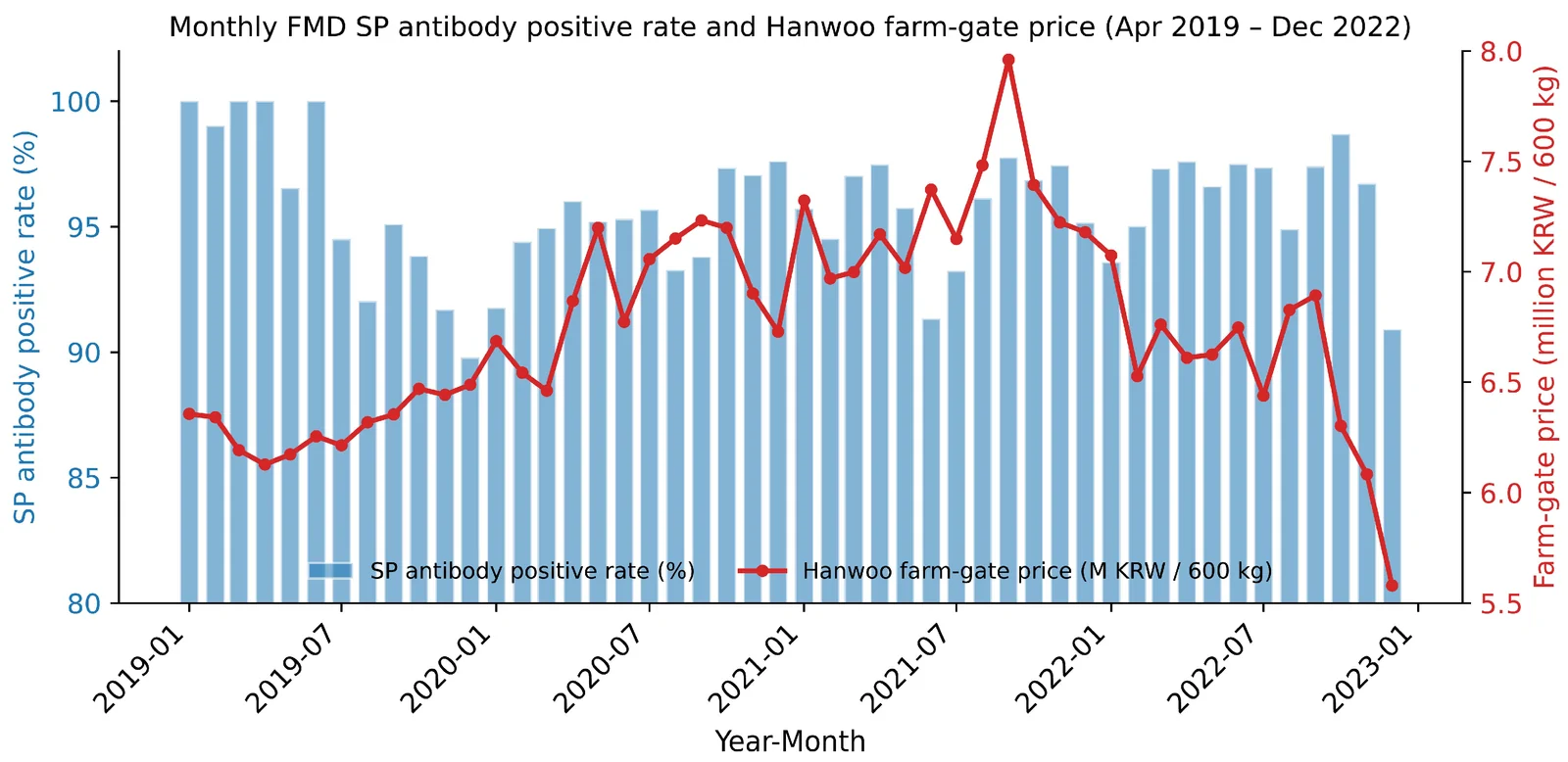
Monthly population-level FMD SP antibody-positive rate (bars, left axis) and Hanwoo farm-gate price (line, right axis) during the analytical period, April 2019–December 2022. The two series are superimposed for visual comparison and correspond to separate y-axes.

The time-lag cohort comprised 75,408 Hanwoo cattle tested over 45 months. The weighted overall SP antibody-positive rate was 96.31%, and the mean of the monthly population-level positive rates was 95.51% (range, 89.79–100.00%). Animals had received a mean of 2.89 cumulative vaccinations and were sampled a mean of 99.6 days after their most recent recorded vaccination. The mean farm-gate price was KRW 6.76 million per 600 kg live animal (Table 1). The monthly trajectories of SP seropositivity and farm-gate price are shown in Figure 1.

### 3.2. Factors Associated with SP Antibody-Negative Status

In the 2022 risk factor cohort (*n* = 131,805), 2276 cattle (1.7%) were SP antibody-negative. Cattle from commercial farms (≥50 head) had modestly higher odds of antibody negativity than those from smaller farms (adjusted OR 1.148, 95% CI 1.047–1.260; *p* = 0.003). Each additional recorded vaccination was associated with lower odds of negativity (adjusted OR 0.886 per dose, 95% CI 0.875–0.896; *p* < 0.001). Sampling at stud farms was also associated with lower odds of negativity than on-farm sampling (adjusted OR 0.551, 95% CI 0.389–0.781; *p* < 0.001) (Table 2).

### 3.3. Time-Lagged Association Between Farm-Gate Price and Seropositivity

**Figure 2:**
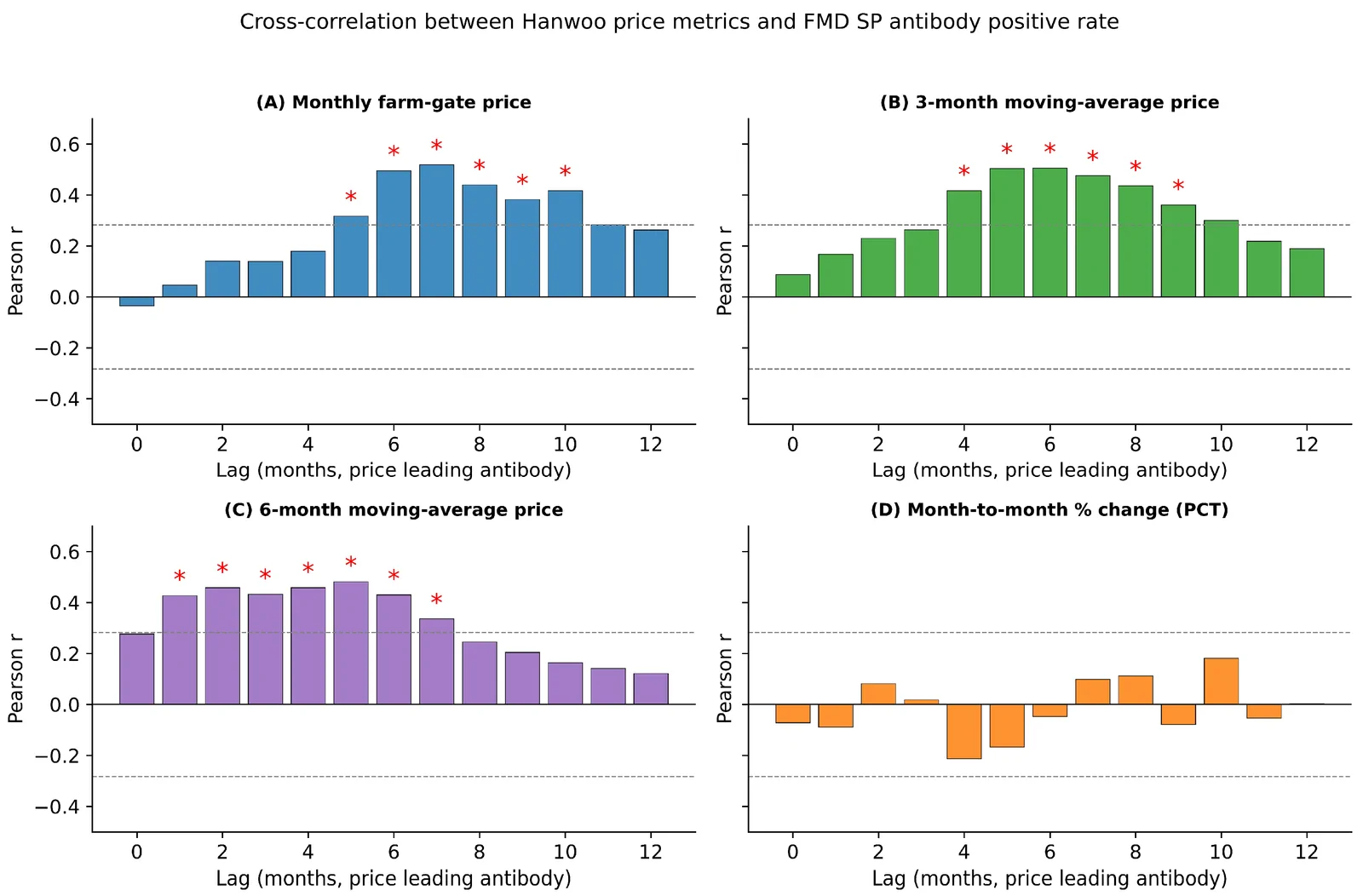
Cross-correlation between Hanwoo farm-gate price metrics and the monthly population-level FMD SP antibody-positive rate at lags 0–12 months: (**A**) monthly price, (**B**) 3-month moving-average price, (**C**) 6-month moving-average price, and (**D**) month-to-month percentage change. Positive lags indicate that price preceded the antibody outcome. Dashed lines indicate approximate 95% confidence bounds; red asterisks denote *p* < 0.05.

Cross-correlation analysis showed a positive, lag-dependent association between farm-gate price and subsequent SP seropositivity (Figure 2; Table 3). For the monthly price series, statistically significant correlations emerged at lag 5 and reached a maximum at lag 7 (r = 0.519, *p* < 0.001). The MA3 price showed its maximum correlation at lag 6 (r = 0.506, *p* < 0.001). The MA6 price produced a broader pattern of positive correlations, with a maximum at lag 5 (r = 0.482, *p* < 0.01). In contrast, PCT was not significantly correlated with seropositivity at any lag (all |r| < 0.22; *p* > 0.17).

### 3.4. Magnitude and Candidate Price Breakpoint

**Figure 3:**
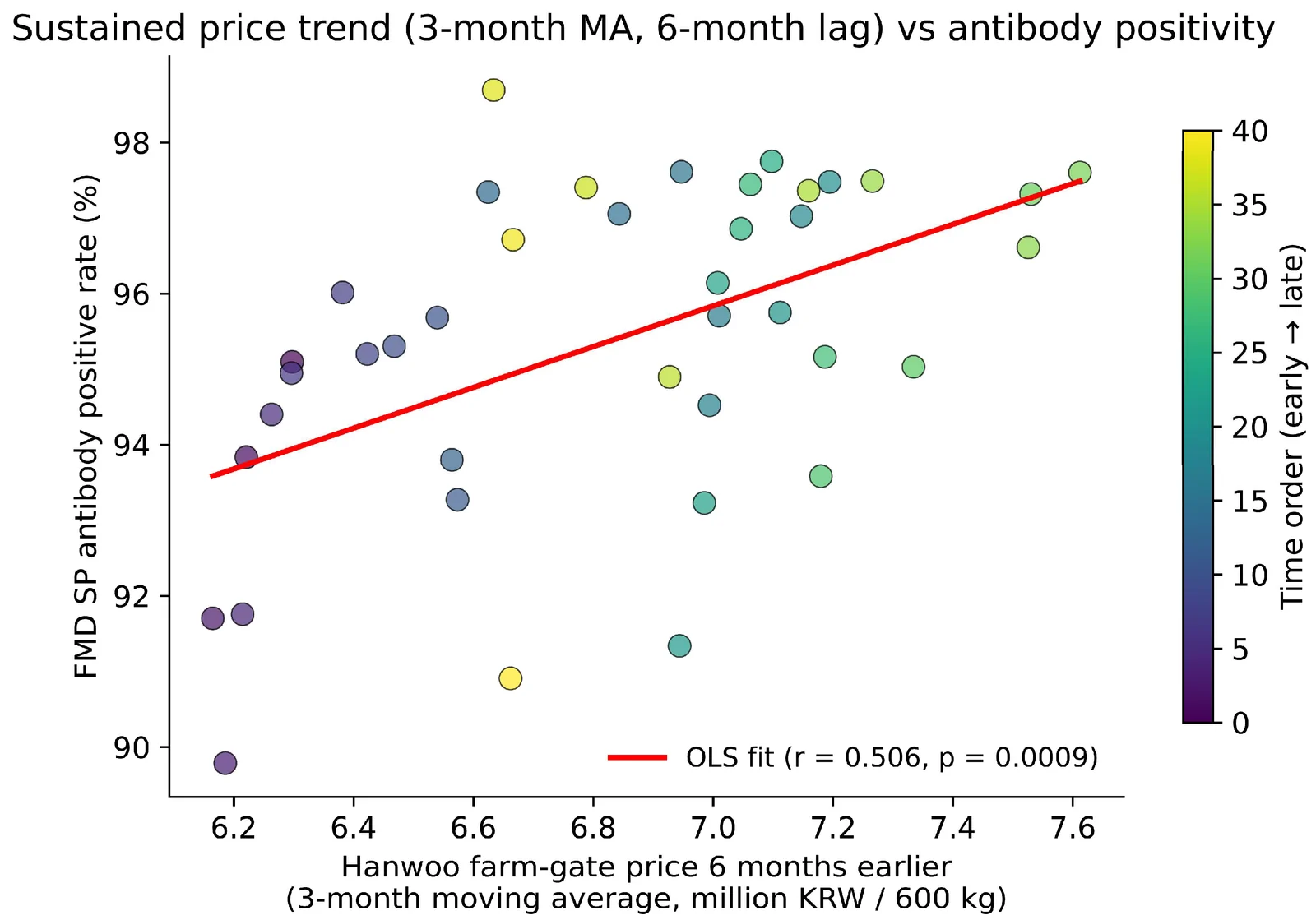
Relationship between the 3-month moving-average Hanwoo farm-gate price 6 months earlier and the monthly population-level FMD SP antibody-positive rate. Point color represents chronological order from early to late observations; the red line is the ordinary least-squares fit.

**Figure 4:**
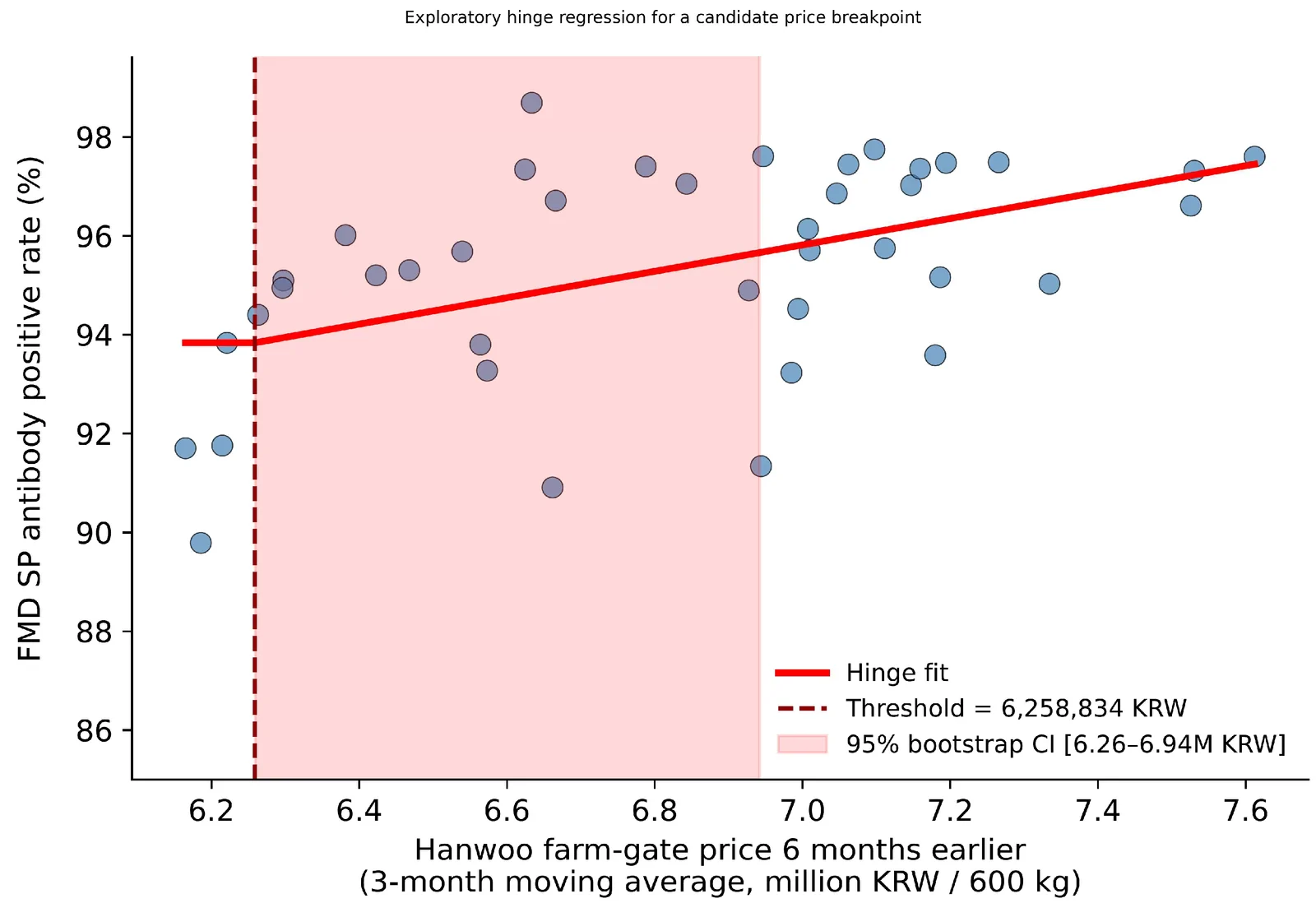
Exploratory hinge regression of monthly population-level FMD SP antibody seropositivity on the 3-month moving-average Hanwoo farm-gate price 6 months earlier. Blue dots represent individual monthly observations. The dashed vertical line marks the estimated breakpoint, and the shaded region indicates its 95% bootstrap confidence interval.

In the linear model, a KRW 100,000 increase in the MA3 farm-gate price measured 6 months earlier was associated with an approximately 0.27-percentage-point increase in monthly SP seropositivity (*p* < 0.001; Figure 3). The exploratory hinge model identified a candidate breakpoint at approximately KRW 6.26 million per 600 kg live animal (95% bootstrap CI, KRW 6.26–6.94 million). Below the estimated breakpoint, the fitted positive rate remained near 94%, whereas above it the fitted rate increased with price (Figure 4). Because this breakpoint was estimated from the same 45-month series, it should be interpreted as hypothesis-generating rather than as a validated intervention threshold.

## 4. Discussion

This nationwide analysis identified a coherent temporal association between sustained Hanwoo farm-gate prices and subsequent FMD SP seropositivity. Three findings were notable. First, monthly price and moving-average price metrics were positively associated with later seropositivity, with the strongest signals at 6–7 months. Second, month-to-month price changes were not associated with the antibody outcome, suggesting that persistent market conditions were more informative than isolated price changes. Third, the cattle-level analysis showed that commercial farm status and the recorded number of vaccinations were independently associated with antibody-negative status. Together, these results support considering economic context as one component of post-vaccination surveillance, while stopping short of attributing the observed serological variation directly to price-driven farmer behavior. Here, the term “contextual signal” denotes a population-level market condition that temporally co-varied with a surveillance outcome; it does not imply that individual farmers changed vaccination behavior because of price.

The 6–7-month lag is operationally plausible in the context of Korea’s recurring vaccination program, in which routine boosters are administered at approximately 6-month intervals [4,5]. Producers are unlikely to reorganize preventive practices in response to a single monthly price movement; sustained conditions may instead influence cash flow expectations, staffing, scheduling, and attention to procedures over several production cycles. This interpretation is consistent with evidence that disease control decisions reflect perceived risk and expected efficacy [7,8,9,10,11,12,13,14,15], while economic constraints and access to veterinary information and services affect sustained implementation [16,17,18,19,20]. International findings provide useful context: behavioral determinants of FMD control intentions and veterinary-vaccine adoption have been studied in Ethiopia [9,19], biannual FMD vaccination has been evaluated as a farm-level economic decision in South Vietnam [16], and vaccination participation and veterinary-service delivery have been examined in India and Kenya [17,20]. These studies differ from the present analysis in design and outcome but collectively support viewing vaccination implementation as embedded within economic and service delivery contexts. The present study extends that perspective by examining routinely reported livestock output prices rather than farmer survey measures. The absence of an association with month-to-month percentage change is therefore informative: the signal was linked to the level and persistence of the market environment rather than to short-lived volatility. Nevertheless, the present design cannot distinguish a behavioral lag from other shared temporal processes, and the correspondence with the booster interval should be regarded as a plausible interpretation rather than proof of mechanism.

The recent FMD literature further supports distinguishing the biological efficacy of a vaccine from the effectiveness of a vaccination program under field conditions. A 2024 systematic review and meta-analysis of routine FMD vaccination in Africa found an overall protective effect but substantial heterogeneity among studies, emphasizing that vaccine performance depends on how vaccination programs are planned and implemented [35]. Field studies also continue to identify non-biological constraints. In northern Lao PDR, resource limitations, awareness, biosecurity, and financial constraints have been recognized as barriers to FMD control [36]; in South Africa, a 2025 study identified persistent gaps in communal farmers’ knowledge even within an FMD protection zone where vaccination is practiced [37]. More recently, participatory modeling of endemic FMD in Indonesia emphasized that vaccination success requires not only vaccine availability but also effective delivery systems and farmer engagement [38]. Although these settings differ markedly from Korea’s mandatory, high-coverage program, they reinforce a common principle: measured population immunity is the downstream result of biological, logistical, informational, and management processes rather than vaccine availability alone.

Several mechanisms could plausibly connect sustained cattle market conditions with vaccination performance, but none were measured directly in this study. Lower output prices may coincide with tighter operating margins, greater sensitivity to labor and service costs, postponement of routine herd procedures, or competing management priorities; conversely, favorable prices may support greater use of external veterinary services, equipment maintenance, and more structured herd management. Price may also co-vary with factors unrelated to vaccination behavior, including herd turnover, input costs, disease expectations, or broader macroeconomic conditions. For this reason, the temporal association should be understood as a surveillance-level relationship rather than evidence for any single behavioral pathway. The lack of association with month-to-month price change, together with stronger signals for the price level and moving averages, is compatible with a process that operates over sustained conditions, but it does not distinguish management responses from other shared temporal influences.

The modestly increased odds of antibody negativity in cattle from commercial farms may reflect greater logistical complexity rather than weaker commitment to disease prevention. Larger operations may depend on batch handling, delegated injections, hired labor, and more complicated scheduling, each of which can introduce variation in vaccine storage, animal restraint, dose delivery, or record keeping. Field studies have linked implementation quality to farm organization and perceived practicality [13,14,24,25,26,30,31], as well as training, infrastructure, and veterinary engagement [21,22,23,27,32]. At the same time, herd size is not universally associated with poorer management; several studies have reported better infrastructure or preventive practice scores in larger farms [26,27]. Accordingly, the adjusted OR of 1.15 should be interpreted as a small population-level difference within this dataset, not as evidence that commercial farms are intrinsically less compliant. Importantly, the time-lag cohort was restricted to commercial farms (≥50 head), whereas the farm size association was estimated in a separate 2022 cattle-level cohort. The current data structure therefore did not permit a valid test of a farm size × price interaction.

The inverse association between cumulative vaccination and antibody-negative status is biologically and programmatically credible and supports the use of repeated immunization records in interpreting SP results. However, SP seronegativity cannot identify the reason for an inadequate response. In addition to missed vaccination, inadequate cold-chain management, poor injection technique, unsuitable handling facilities, the vaccination-to-sampling interval, and individual immune variation may all contribute [4,6,21,22,23,27,33]. This distinction is important because the appropriate intervention differs by cause: reminders may address missed scheduling, whereas training, refrigerator monitoring, restraint support, or review of sampling timing may be needed when vaccination was recorded but immunity remains insufficient. Thus, the market price signal should be interpreted as a contextual indicator of periods warranting closer review, not as a diagnostic marker of non-vaccination.

The candidate breakpoint near KRW 6.26 million per 600 kg animal provides an operationally intuitive hypothesis, but it is not a validated decision threshold. A sustained decline toward this range could prompt low-cost supportive actions, such as targeted reminders, veterinary outreach, cold-chain checks, or intensified serological monitoring, rather than punitive measures. Behavioral and implementation studies indicate that trusted veterinary advice, feasible delivery arrangements, and well-designed incentives can improve the uptake of preventive practices [14,20,24,25,26,32]. However, the estimated breakpoint may shift with inflation, feed costs, subsidy policies, and structural changes in the Hanwoo industry. Although the broader lagged association was observed across several related price metrics, this does not establish the robustness of the specific KRW 6.26 million breakpoint. We did not refit the hinge model across every alternative lag and moving-average specification. The reported bootstrap CI therefore reflects sampling uncertainty within the selected model and does not capture model selection or multiplicity uncertainty. The breakpoint was derived and evaluated in the same short time series and should therefore remain hypothesis-generating until prospectively validated.

From a surveillance perspective, the potential value of price data lies in their availability rather than in their specificity. Farm-gate prices are reported continuously and at low additional cost, whereas direct measurement of vaccination practice, labor availability, service use, or cold-chain quality requires dedicated field data collection. A practical future approach would be to evaluate market indicators jointly with established surveillance information—such as recent farm-level antibody results, vaccination history, herd size, and regional disease activity—to determine whether they improve prediction of periods or populations with suboptimal immunity. Such a framework would use economic indicators to prioritize communication, field support, or targeted monitoring rather than to classify individual farms as compliant or non-compliant. The present findings provide a rationale for testing that integrated approach but not for replacing serological surveillance or applying the estimated breakpoint as an automatic trigger.

This study has several limitations. The time-lag analysis was ecological and used nationally aggregated monthly variables, so it cannot establish that individual farmers changed vaccination behavior in response to price. The 45-month series limited the precision of lag and breakpoint estimates and may have been affected by autocorrelation, seasonality, and common secular trends. The study period also substantially overlapped with the COVID-19 pandemic. We did not include a separate pandemic-period effect in the model, and the available time series provides limited leverage for separating such an effect from other temporal changes. We therefore cannot rule out that pandemic-related changes in labor availability, veterinary-service access, supply chains, or market demand contributed to both farm-gate prices and vaccination-related outcomes. Multiple lag correlations were examined without formal multiplicity adjustment. In addition, months with very different sample sizes contributed equally to the cross-correlation analysis; therefore, seropositivity estimates from months with relatively few tests were less precise and could have greater influence if they were unusually high or low. A sample-size-weighted correlation or a binomial time series model would provide useful sensitivity analyses but was not part of the present study. Farm identifiers, regional price variation, feed costs, labor indicators, and direct measures of vaccination practice were unavailable. Despite these constraints, the study used a large national serological dataset, clearly separated cattle-level and ecological analyses, evaluated several related price metrics, and demonstrated a temporally ordered pattern that was consistent across monthly and moving-average price measures. Future studies should link repeated farm identifiers to regional market and input-cost data, incorporate direct measures of farmer vaccination practice and service access, apply weighted longitudinal and multivariable time series models, and prospectively test whether price-informed support improves vaccination performance.

## 5. Conclusions

In Korea’s mandatory FMD vaccination system, sustained Hanwoo farm-gate prices were positively associated with population-level SP seropositivity 6–7 months later, whereas short-term price changes were not. Commercial-farm status and fewer recorded vaccinations were also associated with antibody-negative results. Farm-gate price should not be interpreted as a direct cause of vaccination failure or as a stand-alone intervention threshold. The term contextual signal is used deliberately: the present ecological analysis does not show that individual farmers changed vaccination behavior in response to price. Nevertheless, routinely available price information may help identify periods when additional communication, implementation support, and serological monitoring could be valuable. Prospective validation should use repeated farm-level data or longitudinal surveys that link vaccination practices, labor and service conditions, and economic exposure before the candidate breakpoint is used for decision making.

## Figures and Tables

**Table 1:** Descriptive characteristics of the commercial Hanwoo time-lag cohort.

Characteristic	Value
Total tested cattle	75,408
Study period	April 2019–December 2022 (45 months)
Mean (SD) tests per month	1676 (2636)
Overall SP antibody-positive rate	96.31%
Mean monthly population-level positive rate	95.51%
Range of monthly positive rate	89.79–100.00%
Mean (SD) cumulative vaccinations per animal	2.89 (0.96)
Mean (SD) days from most recent vaccination to test	99.6 (63.2)
Mean (SD) farm-gate price, KRW/600 kg	6,755,545 (459,201)
Farm-gate price range, KRW/600 kg	5,579,955–7,960,684

Note: The cohort was restricted to Hanwoo cattle from commercial farms (≥50 head) sampled on-farm, with ≤4 cumulative vaccinations and ≥14 days between the most recent vaccination and testing.

**Table 2:** Multivariable logistic regression for SP antibody-negative status in the 2022 cohort (*n* = 131,805).

Variable	Adjusted OR (95% CI)	*p*-Value
Cumulative vaccinations (per additional dose)	0.886 (0.875–0.896)	<0.001
Commercial farm (≥50 vs. <50 head)	1.148 (1.047–1.260)	0.003
Slaughterhouse sampling (vs. on-farm)	0.654 (0.410–1.044)	0.075
Stud-farm sampling (vs. on-farm)	0.551 (0.389–0.781)	<0.001
Days since most recent vaccination (per day)	1.000 (1.000–1.001)	0.137

Abbreviations: CI, confidence interval; OR, odds ratio. Reference categories were farms with <50 head and on-farm sampling.

**Table 3:** Pearson cross-correlation coefficients between Hanwoo price metrics at lag k and monthly population-level FMD SP antibody seropositivity.

Lag (Months)	Monthly Price	3-Month MA	6-Month MA	PCT
0	−0.034	0.088	0.277	−0.072
1	0.047	0.168	0.428 **	−0.089
2	0.141	0.230	0.459 **	0.082
3	0.139	0.263	0.433 **	0.018
4	0.180	0.417 **	0.458 **	−0.213
5	0.318 *	0.504 ***	0.482 **	−0.167
6	0.496 ***	0.506 ***	0.431 **	−0.047
7	0.519 ***	0.476 **	0.336 *	0.099
8	0.440 **	0.436 **	0.246	0.112
9	0.382 *	0.361 *	0.204	−0.078
10	0.417 **	0.300	0.164	0.181
11	0.283	0.218	0.141	−0.053
12	0.263	0.189	0.122	0.004

* *p* < 0.05; ** *p* < 0.01; *** *p* < 0.001. MA, moving average; PCT, month-to-month percentage change.

## Data Availability

Restrictions apply to the availability of the surveillance data. These data were obtained from the Animal and Plant Quarantine Agency (APQA) and may be made available by the corresponding author only with permission from APQA. The monthly farm-gate price data are publicly available from the Korea Institute for Animal Products Quality Evaluation (KAPE) and the Korea Agricultural Marketing Information Service (KAMIS).
